# How should the healthcare system support cancer survivors? Survivors’ and health professionals’ expectations and perception on comprehensive cancer survivorship care in Korea: a qualitative study

**DOI:** 10.1186/s12885-023-11736-y

**Published:** 2023-12-20

**Authors:** Su Jung Lee, Dal-Lae Jin, Young Ae Kim, Hyun-Ju Seo, Seok-Jun Yoon

**Affiliations:** 1 College of Nursing, Institute of Health Science Research, and Inje Institute of Hospice & Palliative Care (IHPC), Inje University, Busan, South Korea; 2 Department of Public Health, Graduate School of Korea University, Seoul, South Korea; 3 Transdisciplinary Major in Learning Health Systems, Graduate School, Korea University, Seoul, South Korea; 4 Division of Cancer Control and Policy, National Cancer Center, Goyang, South Korea; 5 College of Nursing, Chungnam National University, Daejeon, South Korea; 6 Department of Preventive Medicine, Korea University College of Medicine, Seoul, South Korea; 7 Institute for Future Public Health, Graduate School of Public Health, Korea University, Seoul, South Korea

**Keywords:** Cancer survivorship, Cancer survivors, Health professionals, Survivorship care, Qualitative study

## Abstract

**Background:**

Qualitative research on cancer survivors’ need for comprehensive cancer survivorship care within the health care system is limited. Our study aimed to understand cancer survivors' and health professionals' expectations and perceptions for developing a comprehensive cancer survivorship care system in South Korea.

**Methods:**

An exploratory qualitative study was conducted. A total of 16 subjects (11 cancer survivors and 5 health professionals) were purposively sampled from Regional Cancer Survivorship Centers or Cancer Survivor Clinics in Korea. In-depth semi-structured online or face-to-face interviews were conducted. Six steps of thematic analysis were used to analyze data.

**Results:**

The following four primary themes emerged from the interviews: 1) introducing a customized follow-up care system to improve continuity of survivorship care, 2) implementing educational strategies for both survivors and health professionals to manage changed health, and 3) accepting cancer survivors as companions. These three themes included a total of nine subthemes. As a result, the comprehensive survivorship model identified needs in terms of 1) changes in the medical healthcare system and core services that can accommodate the cancer survivors’ condition and 2) necessary care services and social support for cancer survivors.

**Conclusions:**

This study identified the existing gaps in Korea’s current healthcare system regarding comprehensive cancer survivorship care for cancer survivors. Further research on eHealth-based counseling and educational support, the payment models of cancer survivorship care within universal health coverage, and changing social perceptions to strengthen the biopsychosocial needs of cancer survivors is needed.

## Background

Cancer survival rates are considerably increasing in most countries because of technological advances in early detection and effective treatment [1]. People with a cancer diagnosis are termed “cancer survivors” [2]. Cancer survivors need to effectively manage their daily lives, including physical activity and life-long infection prevention, to maintain treatment outcomes. Many cancer survivors strive to return to their everyday lives after a cancer diagnosis; however, cancer treatment causes a multitude of physical, emotional, and practical issues for individuals during and after treatment [3, 4]. Thus, for several cancer survivors, cancer becomes a chronic disease due to the debilitating long-term side effects of its treatment [5, 6].

The fast-growing population of cancer survivors has spotlighted post-treatment needs and issues related to physical, psychological, and social well-being [7–10]. Cancer survivorship focuses on the individual’s health, health behaviors, and well-being; it considers disease recurrence, treatment-related physical symptoms, psychosocial problems, economic impact, and quality of life [11]. More than half of adult cancer survivors reportedly experience work capacity, functional limitations, and persistent physical symptoms that increase healthcare use and cost [2, 6]. Patients with cancer often experience more shock, fear, uncertainty, and loss of control than patients with other diagnoses [12]. Furthermore, cancer survivors may experience unmet supportive care needs such as limited consultation hours with health care providers [13], increased medical costs [14], and the inability to receive treatment closer to home owing to centralized care [15]. Therefore, high-quality survivorship care requires a comprehensive survivorship care delivery system.

Globally, efforts to develop survivorship care models and programs to meet the complex needs of survivors and improve the quality of follow-up care during cancer survivorship are ongoing [16–18]. The 2005 IOM (Institute of Medicine) report on the importance of cancer survivorship care stipulated the provision of care focused on detecting recurrent or secondary cancer, health promotion, surveillance, and management of late side effects [17]. The report further highlighted the application of survivorship care plans to support communication and continuity of care between survivors and service providers [17]. In Canada, a national cancer strategy for survivorship care was launched in 2007, which identified critical priorities for survivorship strategies [19]. The United Kingdom established the National Cancer Survivorship Initiative in 2007 to provide personalized survivorship care focused on recovery [20]. Several models have been proposed in the United States [21]; for example, in the shared care model, cancer specialists and primary care physicians conjointly manage patient care according to the patient’s needs at each stage [22]. Primary cancer survivorship care models, such as the shared care model, referral of survivors to separate survival clinics, or transferring responsibility for survivor care to primary care, have been reported to be equally effective at delivering survivorship care [11].

However, most studies were conducted in Western countries; hence, studies on survivorship care models and plans for Asian cancer survivors are limited. Furthermore, the expectations for cancer survivorship care in Asian countries may differ from those in Western countries since people’s perceptions are often shaped by their culture and society [23]. Contextual and historical circumstances contribute to a person’s attitude toward health, behaviors, and disease experiences [24, 25]; therefore, cultural differences can influence how cancer survivors manage their health [26]. Moreover, although adequate and timely support for survivors is emphasized as an important facilitator for high-quality survivorship care, evidence on the expectations and perceptions of comprehensive survivorship care from the perspective of cancer survivors and health professionals is relatively rare in Asian healthcare systems. Studies on the following topics were conducted in Asia: health professionals’ perception of cancer survivorship care models in Korea [27], cancer survivors’ unmet needs and psychosocial assistance in Korea [28], cancer survivors’ psychological, physical, and information needs in China [29, 30], and community-based shared survivorship care model in Singapore [31].

In Korea, the 5-year (2016–2020) relative survival rate (71.5%) of patients diagnosed with cancer is steadily improving [32]. Since 2018, the number of cancer survivors in Korea has exceeded 2 million, of which approximately 60.1% survived for > 5 years [32]. In response to the growing burden of cancer, the Korean National Cancer Center, a government-funded organization dedicated to cancer research, education and training, and patient care, was established in March 2000 [33]. Furthermore, the Korean government has designated local university hospitals as Regional Cancer Centers (RCCs) to overcome the regional inequality in cancer treatment that has deepened owing to the lack of cancer treatment resources in cities and rural areas, excluding the metropolitan area [34]. Currently, the Central Cancer Survivorship Center is developing a standardized cancer survivor integrated support program, and the National Cancer Center and 13 RCCs are providing integrated support services (e.g., psychiatric oncology, rehabilitation medicine, pain medicine, nutrition management, and social welfare) in the community [34–36]. However, because various medical institutions, including the RCCs, provide cancer survivor management services on their own, the quality of services varies due to differences in workforce, facility, and standards. Moreover, the number of newly diagnosed patients with cancer, cancer prevalence, and cancer survival rate continue to increase. Under these situations, studies have been conducted in Korea on the needs of national cancer survivorship care management [35–38]. This study seeks to provide the empirical evidence needed to build a national cancer survivorship management model through the experience of the pilot project currently in progress. Therefore, a national cancer control strategy with a comprehensive cancer survivorship care management system needs to be planned and implemented to keep pace with the changing Korean cancer statistics, improve the quality of cancer survivor management services, and promote the efficient use of medical resources.

### Aim

This qualitative study aimed to elaborate and understand the expectations and perceptions of comprehensive national cancer survivorship care among cancer survivors and healthcare providers in the Korean context, where a national pilot project to support cancer survivors was recently launched.

## Methods

An exploratory qualitative study design was used. Individual semi-structured interviews were conducted with physicians, care practitioners, and cancer survivors from January 2022 to August 2022. A thematic analysis [39] was used to analyze the data. The descriptive qualitative methodology was used to gain insight into the target phenomenon by excluding a priori conceptualization and providing an accurate description [40]. This study conformed to the Consolidated Standards for Qualitative Research Reporting (COREQ) guidelines [41].

### Participants

Eleven cancer survivors and five health professionals were recruited from National and Regional Cancer Survivorship Centers or Cancer Survivor Clinics to explore the expectations and perceptions of survivors and health professionals for developing a comprehensive cancer survivorship care system. Recruitment of subjects was conducted at each institution, where the researcher explained the purpose of the study and agreed to participate in the study. The cancer survivors had completed treatment for breast, cervical, colorectal, thyroid, and endometrial cancers, brain tumor, Hodgkin’s lymphoma, rhabdomyosarcoma, and acute leukemia. Physicians (family medicine and pediatricians) and care practitioners (social worker and clinical psychologist) directly involved in the care of cancer survivors while working in a hospital or RCC were eligible to participate in the study. Cancer survivors who had received care at a local RCC or who needed continuous post-treatment care in the community were selected. Purposive sampling was used to understand cancer survivors’ and health professionals’ expectations of comprehensive social support in the community [42]. The criteria for selecting study subjects were those with experience in cancer survivor services and who responded to the interview. The number of RCCs has gradually increased; currently, 13 RCCs, including the National Cancer Center, are operating in the region. Following the purposive sampling method of qualitative research, we selected health professionals from two regions (Seoul and Daejeon) and cancer survivors from five regions (Seoul, Daejeon, Gyeonggi-do, Incheon, and Busan) who agreed to participate in the study and had extensive experience to conduct the most appropriate interviews for the study.

### Data collection

Data were collected through in-depth semi-structured interviews lasting between 60 and 120 min. Face-to-face interviews were conducted with the health professionals at their hospitals. Face-to-face and online (via Zoom) interviews were conducted with the cancer survivors. In order to establish a national comprehensive survivorship model for cancer survivors, it was necessary to confirm the care provision system, so it was required to verify not only the experiences of cancer survivors but also the experiences of providers. Researchers with extensive experience conducting qualitative interviews with no direct relationship with the health professionals conducted the interviews. The interviews were structured using a semi-structured interview guide with broad, open-ended questions to ensure consistency (Table 1). The interview guide was developed based on expert opinions and a literature review related to cancer survivor research in Korea and was finalized through pilot interviews with two cancer survivors. Topics discussed during the interview include: 1) comprehensive survivorship care perceived by cancer survivors after cancer treatment, 2) comprehensive survivorship care expected by cancer survivors in the community, and 3) health professionals’ perceptions and expectations of comprehensive survivorship care for survivors. The survey questionnaire was distributed to the study participants in advance to ensure that they were familiar with the content and to recollect their experiences before the interview because of the broad scope of delivery of care and payment system in health policy. Participants were encouraged to describe their experiences with examples. The interviewer could elicit additional information, if needed, via an open question. The sample size of the current study was determined by data saturation [43]. Participants were continuously invited for interviews until no new information was retrieved on the topics covered by the interview guide, and no new topics were identified [43]. Data saturation occurred after conducting 16 interviews.

**Table 1:** Main interview guide for cancer survivors and health professionals

**Cancer survivors**
Please give examples of your experiences to the below questions:
1. Have your thoughts and behaviors about health changed since you were diagnosed with cancer?
2. How did you deal with the problems that occurred after cancer treatment?
3. Please tell us what you felt during the cancer management process. What community service are you expecting?
4. What kind of comprehensive care support do you expect for your cancer management? (e.g., health care system, education, finance, counseling, etc.)
5. How are you managing side effects and managing your health after cancer treatment? What was most challenging about managing your health?
6. What changes do you think are necessary in the community for continued healthcare support for patients with cancer? What services would you like the government to support?
**Health professionals**
Please give examples of your experiences to the below questions:
1. What are the problems cancer survivors face?
2. What care services do you provide to cancer survivors?
3. What kind of cancer survivorship model or care support programs are needed for cancer survivors to manage their health effectively?
4. Considering the current Korean context, what needs to be improved in providing necessary survivorship care to cancer survivors?

### Data analysis

All in-depth interviews were audio or video-recorded, and designated research team members transcribed the interviews verbatim. Information that could be identified from the interview materials was removed to ensure the confidentiality of study participants. The collected data were analyzed by two authors (S.J.Lee and, H.J. Seo) who were experienced and trained in qualitative research methods. Six steps of thematic analysis [39] were conducted using the biopsychosocial model, where biology, psychological, and social factors interconnectedly influence health [44, 45]. These aspects are examined explicitly in studies on comprehensive cancer survivorship. In the first step, to become familiar with the data, we immersed ourselves in the data by reading, re-reading, and repeating the transcribed material. In the second step, we created initial codes by categorizing and flagging important statements. In the third step, the content of the theme was derived by looking at the contents of the code to find meaning. In the fourth step, the researchers reviewed and revised the themes we derived. In the fifth step, the content was refined into core themes, and sub-themes and themes were named. In the final, sixth step, the entire coding tree and results were organized and described along with the main contents by themes. To ensure the trustworthiness of the study, a final consensus was reached through discussions with two qualitative research experts during this process. Each thematic analysis stage was conducted independently by the above two researchers, and consensus was reached after discussion through meetings at each stage. To increase the validity of the analysis, an all-researcher meeting was held regarding the final analysis results to report and review them to increase the reliability of the study.

## Results

Data saturation occurred after 16 interviews (11 cancer survivors and 5 health professionals). Three of the 11 cancer survivors were diagnosed with two or more cancers. Detailed characteristics of cancer survivors and health professionals are summarized in Table 2.

**Table 2:** Participant characteristics

**Characteristic**	*N *(%)	**Mean** (minimum–maximum)
**Cancer survivors (*n* = 11)**		
Gender		
Women	10 (91.0)	
Men	1 (9.0)	
Mean age, years		37 (21–50)
Marital Status		
Married	4 (36.0)	
Unmarried	7 (64.0)	
Cancer type (cancer stage)		
Ovarian cancer (stage IV)	1 (6.6)	
Colorectal cancer (stage I)	1 (6.6)	
Breast cancer		
Stage II	2 (13.0)	
Stage III	1 (6.6)	
Thyroid cancer	2 (13.0)	
Endometrial cancer (Stage III)	2 (13.0)	
Cervical cancer (stage IV)	1 (6.6)	
Brain tumor, Cerebellar astrocytoma	2 (13.0)	
Acute leukemia	1 (6.6)	
Rhabdomyosarcoma (Stage III)	1 (6.6)	
Hodgkin’s lymphoma (Stage III)	1 (6.6)	
Occupation		
Not employed	6 (55.0)	
Business or office worker	2 (18.0)	
On leave	1 (9.0)	
Student	2 (18.0)	
Region		
Seoul	2 (18.0)	
Daejeon	3 (27.0)	
Gyeonggi-do	3 (27.0)	
Incheon	1 (9.0)	
Busan	2 (18.0)	
**Health professionals (*n* = 5)**		
Gender		
Men	3 (60.0)	
Women	2 (40.0)	
Age, years		
30–40	1 (20.0)	
40–50	1 (20.0)	
50–60	3 (60.0)	
Region		
Seoul	2 (40.0)	
Daejeon	3 (60.0)	
Role		
Physician (family physician, pediatrician)	3 (60.0)	
Clinical psychologist	1 (20.0)	
Mental health social worker	1 (20.0)	

**Fig. 1:**
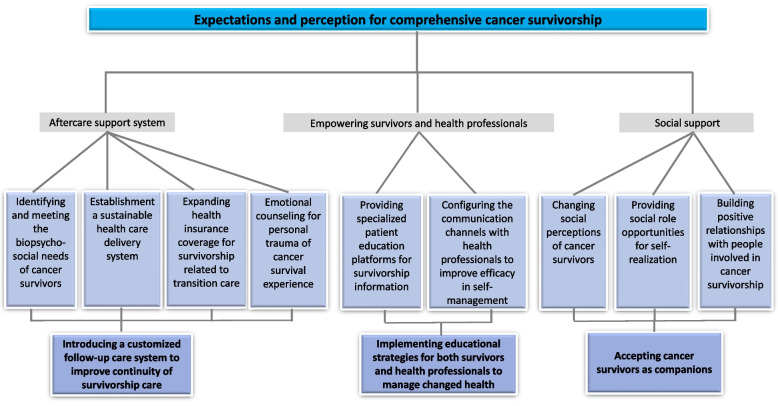
Coding tree for qualitative analysis

The primary themes were as follows: (1) introducing a customized follow-up care system to improve continuity of survivorship care, (2) implementing educational strategies for both survivors and health professionals to manage changed health, and (3) accepting cancer survivors as companions. We have described the expectations and perception of comprehensive cancer survivorship care as evidence of the need for a healthcare system model for cancer survivors in Korea. Figure 1 presents the subthemes and themes according to four categories in a coding tree chart to illustrate the complexity of these expectations and perceptions.

### Theme 1. Introducing a customized follow-up care system to improve the continuity of survivorship care

Cancer survivors and health professionals emphasized the need to establish a sustainable follow-up management system for care needs due to secondary health problems (complications, fear of cancer recurrence [FCR], anxiety, depression, pain, fatigue, etc.) that often occur after cancer treatment such as surgery, chemotherapy, and radiation therapy.

#### Identifying and meeting the biopsychosocial needs of cancer survivors

Several cancer survivors mentioned that continuing care for side effects-induced physical and cognitive health problems, such as pain, lymphedema, and memory, in daily life or mental health problems, such as suicidal ideation and depression, was needed. Health professionals suggested that since the treatment needs of cancer survivors vary according to the cancer type and the time of survival, a multidisciplinary shared care approach could be provided by assessing the care needs of cancer survivors. Both cancer survivors and healthcare providers highlighted not only the biomedical approach addressing the cure and treatment of cancers but also a biopsychosocial approach focusing on identifying and meeting the supportive care needs holistically.

“People like me who have a lot of lymph nodes removed will almost inevitably have lymphedema as a side effect, so I would like people like me to connect with rehabilitation medicine providers. Please share links to where I can get lymphatic massage regularly or how much it costs. I went around five places and found a convalescent hospital that did lymphatic massage with difficulty.” (Cancer survivors #4).

“After treatment, I thought I wanted to die. One day, I happened to see an advertisement for The Regional Cancer Center (RCC) for Cancer Survivors in the newspaper. After receiving aftercare at the center, I gained emotional and psychological stability and comfort.” (Cancer survivor #1).

“In the case of brain tumor, the demand for ‘cognitive function test’ is high.” (Pediatrician #1)

“Depending on the survivors’ care needs, a program for cancer survivors can be provided by bundling care contents into a package, such as exercise, prescription, nutrition, and education.” (Family Physician #2).

“We provide care services in the form of shared care. We are cooperating with other professionals. In patient management, such as recurrence screening, secondary cancer screening, cardiovascular and bone health management, and vaccination, oncologists or surgeons refer patients to us. It is efficient to evaluate mental health-related parts in the Department of Psychiatry.” (Family physician #1).

“The Regional Cancer Centers for Cancer Survivors performs periodic functional evaluations by applying the national evaluation scale and provides programs for cancer survivors.” (Clinical Psychologist #1).

### Establishing a sustainable healthcare delivery system

Cancer survivors desired an inter-connected hospital system to help them receive follow-up care at hospitals closer to their homes. Cancer survivors often experience rejection when visiting local hospitals. However, health professionals proposed that community doctors must be able to deal with complex chronic diseases to manage cancer survivors at local clinics and that patient information must be shared between hospitals through the treatment summary or cancer survivorship care plan covered by national health insurance. However, when cancer survivors visit another hospital other than the hospital where they were originally treated, there are no accessible medical records due to the PERSONAL INFORMATION PROTECTION ACT in South Korea. Moreover, there is no formal and standardized cancer survivorship care plan. Therefore, healthcare professionals, as well as cancer survivors, underlined a sustainable establishment of a cancer survivorship care delivery system connected to the insurance system.

“My house is in Daejeon, not Seoul. If I go to a hospital near my house and say that I am a cancer patient, there are times when the hospital itself does not accept it.” (Cancer survivor #1).

“In a situation where it is difficult to go to the hospital where the surgery was performed, it is much better to go to a nearby hospital. So, I copy dozens of medical records and leave them at home. Whenever I go to the hospital, I'm carrying it. So, if I agree that other hospitals can view my medical record data, it would be nice to view it quickly on a computer.” (Cancer survivor #4).

“Currently, there are difficulties in collecting treatment summary (TS) of patients in hospitals. If the TS is organized, it would be nice to have a way to support this summary because the doctor can check when the picture was taken during the treatment process, what problems there were, what medications were used, and when follow-up was required… I propose a care model in which cancer survivors can manage their own health after active survivorship management by writing a care plan for the patient’s follow-up care.” (Family physician #2).

Furthermore, the health professionals pointed out practical problems regarding the current healthcare system.

“In clinical practice, doctors are busy with patient care, so it seems difficult to conduct a multidisciplinary program under the current standards. In order to proceed with the multidisciplinary system, it is possible only when the needs of patients and the needs of hospital managers are met.” (Family physician #2).

“A mutual medical treatment request form is sent from the tertiary general hospital to the primary hospital. When a resident writes a draft and reviews a chart, I look at it and place an order. There may be practical difficulties in proceeding from the standpoint of medical specialists without residents.” (Family physician #1).

“Clinical psychologists play a role only in showing the currently developed psychological programs. Roles are primarily limited to psychological evaluation, individual counseling, and program management.” (Clinical Psychologist #1).

“There are many patients who come from the provinces to large hospitals in Seoul for treatment, and there are disruptions to classes after returning to school; so, remote medical treatment is also necessary.” (Pediatrician #1).

### Expanding health insurance coverage for survivorship related to transitional care

Many cancer survivors stated they needed financial or material support, such as screening costs, medications, stoma products, transportation, and wig rental, to manage their cancer survivorship effectively. Physicians mentioned that participation rates in community primary care are low because managing cancer survivors is difficult and risky and opined that health professionals should be appropriately compensated. In addition, doctors stated that it is practically impossible to conduct 15 min of consultation in patient care in a limited system.

“Extended Health Insurance only covers treatment related to the diagnosed cancer; so, it does not cover cosmetic or complementary treatments or treatment for conditions not related to the diagnosed cancer.” (Cancer survivor # 4).

“Medical support for cancer patients is an extended health insurance period of up to 5 years in the country. So, after 5 years, I have to do a health checkup like the general public, but honestly, the cost is not cheap.” (Cancer survivor # 1).

“It takes a lot of time to create a care plan or treatment summary by checking the cancer treatment history and various chronic disease conditions of a subject who has completed active treatment. Therefore, if the government lowers the barriers related to patient out-of-pocket expenses and rewards medical staff for referred and returned patients in cancer survivor treatment, it can motivate medical staff and lower economic barriers for patients.” (Family physician #1).

“Social workers are currently providing counseling and guidance on education, resource linkage, discharge plans, etc.; but there is no charge for this part.” (Mental health social worker #1).

“For cancer survivors, only the part related to active treatment and postoperative sequelae is covered by 'health insurance.’ The RCC is being operated to support the health promotion of patients who have not relapsed among patients who have completed active treatment. However, it is still necessary to consider an insurance system for the treatment necessary for severely ill patients who cannot come to the center… Because cancer survivors are patients with complex chronic diseases, a high level of judgment and effort are required. However, since the fee is the same, it is difficult for doctors in practice to afford it within the limited time frame” (Family physician #2).

“When receiving psychological counseling, there is no medical fee of clinical psychologists. Therefore, we propose to create a medical fee of’Cancer survivor Counseling education.’" (Clinical psychologist #1).

### Emotional counseling for personal trauma of cancer survival experience

Cancer survivors mentioned desperately needing counseling at one point because they experienced psychological dilemmas such as FCR, depression, social role conflict, and suicidal ideation. In addition, cancer survivors mentioned that counseling from health professionals, including psycho-oncologists, oncology nurses, and psychologists, was needed for themselves and their families or caregivers.

“I was a very strong person. I was a mother, cared for my children, had a job, and had a social life. I had never thought about suicide, but it changed when I was in the situation of a cancer patient. My life was falling apart. At that time, I thought I needed someone to comfort me while talking with me rather than taking psychiatric medication….I have many things to help the children in the future, but because I can't do it, the children feel the fear of their mother's death or the vacancy." (Cancer survivor #1).

“In my first breast cancer diagnosis, the mental part, distress, and things like that were too big. When I was receiving cancer treatment, I couldn't talk about various things because there were waiting patients at the hospital, and I couldn't even talk to the surgeon about my mental health. I was in a state where I didn't know how to talk or to whom when I needed a conversation.” (Cancer survivor #2).

“Mentally, too much trauma has occurred to me, so I still receive counseling and treatment. It's been three years, but since then until now, I think my body and mind have all collapsed to that extent.” (Cancer survivor #3).

“When I was hospitalized at the cancer center, there were a lot of patients but only two medical staff. So, I couldn't have a long consultation and could only talk for about 5 to 10 min. So, I was very disappointed at that time. So, I went to a psychiatric clinic near the neighborhood, thinking it would be possible to receive long-term counseling. Still, since the hospital doesn't know about the difficulties that cancer patients feel, I felt like the counseling was out of the way…My husband was also very shocked. The kids were not at home when I had the surgery, and they saw that I was bald because I lost all my hair, so I thought the kids must have been in shock, too. So, I looked for family counseling, but the cost was too expensive. So, I couldn't do it, but I even thought it would be nice to have family counseling. Because not only me but my family also had a hard time.” (Cancer survivor #4).

### Theme 2. Implementing educational strategies for both survivors and health professionals to manage changed health

#### Providing a specialized patient education platform for survivorship information

Most cancer survivors noted that information on coping strategies or managing health problems after treatment is important because they will have to live with a life-long anxiety of recurrence. However, cancer survivors complained that finding a place that provided necessary education or advice on managing changed health and adapting their lifestyle after acute treatment was challenging. Cancer survivors stated that they received little education related to follow-up care other than that provided during surgery, chemotherapy, radiation therapy (RT) treatment, or drug administration. Therefore, cancer survivors wanted an educational platform where they could find cancer-specific and individualized survivorship information.

“When I was discharged from S Hospital, I learned to exercise through videos; but it didn’t really work out. That’s why I thought practical training like massage was necessary.” (Cancer survivors # 1).

“At the hospital, the treatment was over as if surgery and chemotherapy in cancer care were all. After that, the side effects management is just difficult for the patient to deal with alone. Cancer patients like me have a lot of questions, and I want to get information, so I look for it at cafes and YouTube. But it's difficult to find information, and I don't know if it's accurate.” (Cancer survivor #4).

“If you go to the search portal, many therapeutic drugs are on sale. Like miracle water. There's a lot of information about what works for the most part, but I think the government should help people get it right.” (Cancer survivor #2).

“Because patients have a great fear of recurrence, education on secondary cancer, nutritional counseling, and lymphedema is considered an essential area. For breast cancer patients, education about lymphedema is critical. There is a fee for cancer education, but this is for explaining the prognosis according to surgical treatment, and it is currently difficult to place orders for exercise, prescription, nutrition, and educational counseling.” (Family physician #2).

“In fact, since cancer survivors have high health care needs, providing appropriate information to cancer survivors can help them form evidence-based health promotion habits without being swayed by advertisements for health functional foods or health promotion products… When cancer survivors come to family medicine, they are often the first to hear about a second cancer screening. When patients were provided with information such as proper check-up and treatment for cardiovascular and bone health, vaccination, healthy weight, and cancer treatment-related fatigue, anxious patients responded well.” (Family physician #1).

Furthermore, education and training of health professionals to certify their qualifications for educating cancer survivors were considered necessary factors.

“Local hospital doctors should be able to provide health care for cancer survivors with complex chronic diseases who are at high risk. For example, a local clinic doctor needs qualifications or training to be certified as a healthcare provider capable of managing cancer survivors. “(Family physician #1).

“A multidisciplinary approach is also aware of the ‘necessity’; but there are cases in which there is no specific knowledge of each role on what to do in the field.” (Family physician #2).

### Configuring the communication channels with health professionals to improve efficacy in self-management

Cancer survivors mentioned they often need counseling to help them make decisions about symptoms requiring a visit to the hospital, ask questions, or receive educational information for self-management. However, health professionals stated that the medical situation requires the introduction of an efficient system because resources available for nutritional or exercise counseling for patients are limited. Given the lack of communication channels, such as oncologists or transitional nurse navigators, cancer survivors have difficulty in self-management when confronting symptom management or discomfort.

“It would be nice if a DB was built to know how much swelling is normal and what to do in this case. If I ask a question, the cancer center can answer it. In doing so, patients also share their experiences. It would be nice to have an app to share that experience. Even when receiving chemotherapy, I had severe constipation or numbness in my hands and feet, but I had never consulted at a hospital. When I called the hospital, the only thing I could do was see a doctor in person.” (Cancer survivor #4).

“My house is in the countryside, so going to the hospital in Seoul requires a lot of transportation time and cost, and it's physically difficult. In the case of just looking at my progress, I wish non-face-to-face treatment would be possible.” (Cancer survivor # 10).

“If I have any questions, I call the hospital and ask them. I think it would be helpful to have a system on the homepage or bulletin board where patients can ask questions and share them together.” (Cancer survivors # 8).

### Theme 3. Accepting cancer survivors as companions

#### Changing the social perceptions of cancer survivors

Some cancer survivors desired changes in social understanding and attitudes toward them. They stated that they inform people that they are cancer survivors to improve social awareness of patients with cancer. In other words, they said that there is a need for a change in the social atmosphere or social awareness so that cancer survivors can live like other people without being discriminated against in society.

“Even at that time, no one readily said that I had cancer. Because there were so many negative parts rather than positive perceptions. At work, it was difficult for me to say’I am a cancer patient because I would be disadvantaged, and no one reached out to me first when I said I had cancer.” (Cancer survivor #2).

“I have plans to return to work, but people will pity me and see cancer as a deadly disease. So, I think that not only the patient but also the people around the patient (colleagues, etc.) need an accepting attitude. I think people around cancer patients need to change their perspective so that cancer patients can go back to their daily lives.” (Cancer survivor #4).

“Since I had childhood cancer, I worry a lot about whether it is right to let others know about my medical history of having been treated in the past or whether it is right to hide it. However, I thought that if I set an example that I overcame well, there would be a need to hide it, so I actually told the professors at the university interview. Fortunately, the professors took good care of me, so I think I could enter the university.” (Cancer survivor #10).

### Providing social role opportunities for self-realization

Cancer survivors desired to spend the rest of their lives meaningfully by viewing their cancer diagnosis as an opportunity. They yearned for a chance to participate in society while simultaneously managing cancer to some extent in their daily lives. Cancer survivors stated that they needed social services such as alternative education (e.g., cyber school or e-learning program) and employment information that they did not receive during treatment. As social members, they want to engage in social roles to achieve self-realization.

“I am a cancer patient, and after surgery, I am working as a consultant at a stoma product company. My self-confidence dropped significantly when I underwent surgery such as a stoma, so I wish I could connect people with things that cancer survivors can do.” (Cancer survivor #9).

“I want to get a job again, but I can't. I have a lot of gray hair, but I didn't dye it because I was told not to dye it for health, but appearance is also important to return to society. If society creates job opportunities for cancer patients, I think they will be able to gain confidence in returning to society.” (Cancer survivor # 1).

### Building positive relationships with the people involved in cancer survivorship

Cancer survivors expressed their gratitude for overcoming the treatment process because of the love and support of their families and friends during the treatment process. Some health professionals mentioned the need for systems to support the ongoing support system of cancer survivors by providing respite programs (e.g., leisure services) to families caring for cancer survivors.

“My family helped me a lot. When I was discharged from the hospital after surgery, my parents even cleaned the dust from the cracks in the door. My husband is also educated, so my family pays a lot of attention to hygiene management because uncleanliness lowers my immunity.” (Cancer survivor # 1).

“I hope that a community of cancer patients with the same feelings can be activated and gain information and comfort together.” (Cancer Survivor # 4)

“When I entered my sister’s room, there was a desk full of papers and cancer-related books. I just cried. I was moved! … I thought of myself as a person with little use in this world, but my sister helped me to live again; she's a person I'm really grateful for.” (Cancer survivor #6).

In addition, cancer survivors desired to network with health professionals (oncology nurses or doctors, RCC doctors, clinical psychologists, social workers, etc.) who could provide social support and resource links for information.

“I had a lot of questions, so whenever I went to the hospital, I stopped by the oncology nurse and asked a lot of questions. Sometimes, I called the ward and asked, and they solved the problem. I hope to get the information or help from an expert, whether face-to-face, over the Internet, or the phone.” (Cancer survivor #8).

“Regarding leisure services for children with cancer, the palliative care support team allows parents to take a short break for children with cancer, and various support foundations provide support programs for children with cancer.”(Pediatrician #1).

## Discussion

The results of this qualitative study elicited diverse perspectives from cancer survivors and health professionals on comprehensive cancer survivorship care in the Korean healthcare system. These expectations and perceptions on comprehensive cancer survivorship care highlighted the need for the following aspects within the health care system: empowering a customized follow-up care system to improve continuity of survivorship care, implementing educational strategies to manage changed health, and accepting cancer survivors as companions.

Regarding the follow-up care system, participants emphasized the importance of assessing and mediating the biopsychosocial needs of cancer survivors, having a continuous care delivery system, and establishing a fee or insurance system for cancer survivorship care. Cancer survivors experience lifelong physical, emotional, and social problems [31]. In 2005, the IOM reported the situation of cancer survivors as “lost in transition,” and thereafter, cancer survivorship care models have emphasized facilitating transitional care management to support patients transitioning from active treatment to follow-up care [22]. Our findings suggest that health professionals providing comprehensive survivorship care (e.g., pain, lymphedema management, counseling for suicidal ideation, or job training) should assess the diverse individual needs of cancer survivors with complex chronic diseases to ensure they receive optimal transitional care. In the United States, assessment of various care needs (e.g., level of functional impairment, symptoms of recurrence, presence/absence of risk factors, symptoms of anxiety, and patient preference) is based on recommended guidelines [46]; moreover, a standard cancer treatment guideline recommending that palliative care should be carried out at all stages from the beginning of cancer treatment to advanced cancer stages was published as well [47].

However, in Korea, the current needs assessment and care programs are limited in some RCCs; therefore, this study suggests the need to develop a care reimbursement system (e.g., consultation fee and care plan preparation fee) to ensure that follow-up care can be performed in RCCs and hospitals. Furthermore, previous Korean studies confirmed the unmet psychosocial needs of cancer survivors [28], the lack of shared care or multidisciplinary care system, a poorly customized long-term cancer survivorship care, and the lack of emotional support for cancer survivors owing to insufficient health care professionals were pointed out [27]. In our study, Korean cancer survivors often experienced treatment refusal when they visited local hospitals due to health problems that occurred during follow-up care. Our findings revealed that local hospitals are reluctant to care for cancer survivors because the burden of treatment for cancer patients with complex problems is high, and no special medical fee for treatment is set. The National Cancer Center and RCCs in 13 regions across Korea have attempted to establish integrated support services since 2020; the National Cancer Survivorship Center is currently developing a standardized integrated support program for cancer survivors [33, 34]. Moreover, a recent study on the effect of a cancer care model integrating RCCs and public health centers (PHCs) for accessibility of medical facilities for patients with cancer in rural areas reported positive evaluations from participants [48]. Therefore, a sustainable transitional care system must be established in cooperation with hospitals, public health centers, or RCCs for the follow-up care of cancer survivors in the community.

Furthermore, our study emphasized that “getting financial assistance for coverage of costs” is critical to enabling a customized and sustainable transitional care system for cancer survivors. Our study results confirmed that the cost of preparing a treatment summary (or post-care plan) required for inter-hospital referral of cancer survivors and that of consultations with social workers and clinical psychologists need to be paid. The Oncology Care Model (OCM), launched in the United States in 2016, combines the attributes of the delivery approach of primary care (care coordination, evidence-based guidelines, accessibility, patient-centeredness, and continuous quality improvement) with financial incentives to provide efficient and high-quality care services [49]. This model has a characteristic financial incentive strategy where health professionals may charge an additional monthly fee to help improve care [50]. In our study, cancer survivors desired financial support with the cost of diagnostic tests or medications after the 5-year “Relieved Co-payment Policy” to lower the out-of-pocket expenses and social support (preparation for employment, wigs, transportation expenses for distant medical treatment, etc.). Financial support is an important factor in the healthcare compliance of cancer survivors. Patients who experience financial toxicity (patient-level impact of cancer treatment costs) often skip or delay treatment or medication and forgo mental health treatment [51]. In particular, working-age (age 15– 64 years at the time of diagnosis) cancer survivors may experience considerable financial toxicity, which negatively impacts them [52, 53]. A recent study in a universal Korean care setting confirmed that financial toxicity was associated with FCR, loss of purpose, uncertainty, and hopelessness among working-age cancer survivors [54]. Therefore, the financial readiness of cancer survivors needs to be confirmed at the national level, and welfare benefits must be provided to cancer survivors according to their grades.

Additionally, living with sympathetic people, emotional counseling on cancer trauma, and networking with people (such as oncology nurses, physicians, cancer survivor peer groups, etc.) were perceived as important for cancer survivorship care. Most of the cancer survivors mentioned that they experienced psychological trauma while going through the crisis of a possible sudden death. Previous studies have highlighted the need for psychosocial rehabilitation for cancer survivors [28, 55].

The provision of cancer education for cancer survivors, training for health professionals to strengthen their capabilities, and establishment of efficient communication channels to obtain information from health professionals were expected for an effective cancer survivorship care system. Most cancer survivors mentioned that they are sensitive about their health condition after treatment; hence, they went through a trial-and-error method of referring to books, searching the Internet, or asking others to acquire the needed information. Cancer survivors expressed anxiety and concern about coping with problems while searching for data unaided, and they desired reliable information on managing their health and daily life. Therefore, sufficient counseling and education for cancer survivors, such as post-treatment examinations, lifestyle management, and resource linkage, are vital necessities. The need for education regarding informational support has been emphasized for various cancer survivors in previous studies [56, 57]. In particular, our study mentioned that health professionals need periodic education or training on cancer-related care because cancer survivors are high-risk patients with complex chronic diseases. Currently, Korea provides general educational materials for cancer patients at the National Cancer Center [34]; however, this study highlighted the need for more training for health professionals and dedicated communication channels through which cancer survivors can consult when they require specific information in a decision-making situation. In this regard, a system for counseling and education through various communication channels, such as telephones, hospital web portal bulletin boards, and online platforms, was proposed. Considering that cancer survivors have a positive attitude toward e-health [58], telephones or the Internet can be used as a communication channel between medical institutions and cancer survivors.

Finally, regarding social support, the expectation from cancer survivorship care was that cancer survivors would be accepted as companions and that the social perception of cancer survivors would change and more social role opportunities would be offered. Most cancer survivors in this study were school- or working-age individuals. While recovering their health and resuming their daily life after cancer treatment, they were attempting to return to school or social life or find a new social role. However, cancer survivors expressed concern that society’s recognition of them would be working to their disadvantage. Some cancer survivors in this study attempted to display proactive attitudes, such as revealing or publicizing that they were cancer survivors to change social perceptions. In addition, cancer survivors are expected to be provided with job information, opportunities to participate in social activities, and educational opportunities (online mode) to help prepare them for employment or further studies. However, the social environment for cancer survivors in Korea remains unwelcoming. Many Koreans reportedly have a negative perception of cancer survivors [59], although cancer is the leading cause of death in Korea [60], with a 5-year relative survival rate of 70.3% between 2014 and 2018, and contributed to more than 2 million cases by the end of 2018 [61]. Cancer survivors’ perceptions, such as fear of discrimination, self-blame, and shame, can cause psychosocial and medical problems and contribute to depression, distress, physical and emotional maladjustment, poor quality of life, and reduced treatment adherence [62–64]. Therefore, positive perceptions of cancer survivors need to be promoted through public relations using strategic messages for a comprehensive cancer survivorship care system.

Also, our study results revealed that although the needs of cancer survivors in Korea were almost identical to those of survivors in other countries, some unique differences were perceptible. Even cancer survivors with positive attitudes experience psychological crises such as suicidal ideation, depression, and helplessness after cancer treatment; hence, providing timely counseling according to the evaluation of the psychological state is crucial. In addition, counseling should be conducted for cancer survivors and their families if possible. In particular, because Korea has a more developed family or community culture than other countries, cancer survivors recognized people networks as an important aspect of cancer survivorship care.

This study has some limitations. First, conclusions derived from this study are limited to participants who have experience using RCCs or receiving follow-up care in the Cancer Survivor Clinic. Therefore, the needs of unmanaged cancer survivors after treatment may be much different than those reported herein. Second, although gender or age was not used as exclusion criteria, many current service users who agreed to participate in the interview were female, mostly young, and relatively few general cancer survivors (e.g., lung cancer, stomach cancer). In future research, it is necessary to identify the service needs of cancer survivors of more diverse ages and cancer type. Most study participants are women, perhaps because they are more open to engaging in social activities [60] or sharing their experiences. Therefore, it cannot be assumed that the comprehensive social support experienced by cancer survivors in our study applies to all cancer survivors. Nevertheless, our sample included adults in Korea as cancer survivors of representative cancer types. It was made with a qualitative research method of purposeful sampling that could well interview the aspects of their post-treatment follow-up care process owing to their rich experience. In addition, nurses were not included in this study as participants were healthcare professionals due to not working in the RCC. However, in South Korea, there are oncology nurses who provide nursing services targeted at cancer patients in hospital settings[65, 66]. Moreover, our study findings show that cancer survivors wanted to obtain information and knowledge from experts, so we suggest that it is an important part to consider including oncology nurses in the cancer survivor model in the future. Therefore, the expectations and perception of comprehensive cancer survivorship care within the Korean healthcare system were confirmed in this study. Moreover, this study included multidisciplinary health professionals as participants as well; hence, it was possible to more realistically ensure the experience of survivorship care in the context of the Korean healthcare system. To develop a Korean national cancer survivor care model and to gain a comprehensive understanding of cancer survivors’ service, we needed to identify and gather opinions and suggestions from cancer survivors and health professionals. As a result, cancer survivors mainly expressed diverse transition care needs, and health professionals’ responded by primarily focusing on areas requiring change in the provision of services related to the medical system and core care services. The significance of this study is that these differences and overlaps in opinions indicate the differences between the consumer and provider sides of the cancer survivor management model, which is a service provision model, and suggest that they can be included in comprehensive considerations in future national model development.

## Conclusion

Cancer survivorship care is becoming a worldwide concern as the number of patients with cancer and that of cancer survivors continues to increase [22]. Our exploratory study on the expectations and perceptions of cancer survivors and health professionals on comprehensive survivorship care is a possible source of practical knowledge for developing a high-quality survivorship care system model within the current Korean health care system. Our findings highlight the need to develop a sustainable transitional care system customized to the individual care needs of cancer survivors. In our study, a policy on care reimbursement systems for survivorship care in hospitals is needed in response to the establishment of a sustainable transition survivorship care system. In addition, Korean cancer survivors’ ability to use ICT (Information and Communications Technologies) and survivorship-related networking can be an advantage for survivors’ communication and information acquisition. These results can be used as evidence for formulating future policies for comprehensive survivorship care and providing appropriate national healthcare services for cancer survivors in Korea, thus improving cancer survival rates and quality of life and contributing to the efficiency of national health insurance financial expenses.

## Data Availability

The datasets generated and/or analyzed during the current study are not publicly available due to IRB restrictions but are available from the corresponding author upon reasonable request.

## Abbreviations

FCR: Fear of cancer recurrence
IOM: Institute of Medicine
RCC: Regional Cancer Center
ICT: Information and Communications Technologies
