# Correction: How should the healthcare system support cancer survivors? Survivors’ and health professionals’ expectations and perception on comprehensive cancer survivorship care in Korea: a qualitative study

**DOI:** 10.1186/s12885-023-11788-0

**Published:** 2024-01-02

**Authors:** Su Jung Lee, Dal-Lae Jin, Young Ae Kim, Hyun-Ju Seo, Seok-Jun Yoon

**Affiliations:** 1 College of Nursing, Institute of Health Science Research, and Inje Institute of Hospice & Palliative Care (IHPC), Inje University, Busan, South Korea; 2 Department of Public Health, Graduate School of Korea University, Seoul, South Korea; 3 Transdisciplinary Major in Learning Health Systems, Graduate School, Korea University, Seoul, South Korea; 4 Division of Cancer Control and Policy, National Cancer Center, Goyang, South Korea; 5 College of Nursing, Chungnam National University, Daejeon, South Korea; 6 Department of Preventive Medicine, Korea University College of Medicine, Seoul, South Korea; 7 Institute for Future Public Health, Graduate School of Public Health, Korea University, Seoul, South Korea

**Correction: BMC Cancer 23, 1255 (2023)**

10.1186/s12885-023-11736-y

Following publication of the original article [1], the authors reported a missing grant number in the funding section of their article. The funding section should read as follows: The National Cancer Center of South Korea provided funding to support this work **(grant number: HA21C0206)**; however, it had no role in the design and conduct of the study; collection, management, analysis, and interpretation of the data; preparation, review, or approval of the manuscript; or decision to submit the manuscript for publication.

## References

[1] Lee SJ, Jin DL, Kim YA, et al. How should the healthcare system support cancer survivors? Survivors’ and health professionals’ expectations and perception on comprehensive cancer survivorship care in Korea: a qualitative study. BMC Cancer. 2023;23:1255. 10.1186/s12885-023-11736-y. 10.1186/s12885-023-11736-y PMC10731886 38124040

